# Evaluation of Pressure Capacitive Sensors for Application in Grasping and Manipulation Analysis

**DOI:** 10.3390/s17122846

**Published:** 2017-12-08

**Authors:** Paola Pessia, Francesca Cordella, Emiliano Schena, Angelo Davalli, Rinaldo Sacchetti, Loredana Zollo

**Affiliations:** 1 Unit of Biomedical Robotics and Biomicrosystems, University Campus Bio-Medico of Rome, via Alvaro del Portillo 21, 00128 Rome, Italy; paola.pessia@alcampus.it (P.P.); l.zollo@unicampus.it (L.Z.); 2 Unit of Measurements and Biomedical Instrumentation, University Campus Bio-Medico of Rome, via Alvaro del Portillo 21, 00128 Rome, Italy; e.schena@unicampus.it; 3 Centro Protesi INAIL, Via Rabuina 14, 40054 Budrio (BO), Italy; a.davalli@inail.it (A.D.); r.sacchetti@inail.it (R.S.)

**Keywords:** capacitive pressure sensor, sensor characterization, grasping and manipulation analysis

## Abstract

The analysis of the human grasping and manipulation capabilities is paramount for investigating human sensory-motor control and developing prosthetic and robotic hands resembling the human ones. A viable solution to perform this analysis is to develop instrumented objects measuring the interaction forces with the hand. In this context, the performance of the sensors embedded in the objects is crucial. This paper focuses on the experimental characterization of a class of capacitive pressure sensors suitable for biomechanical analysis. The analysis was performed in three loading conditions (Distributed load, 9 Tips load, and Wave-shaped load, thanks to three different inter-elements) via a traction/compression testing machine. Sensor assessment was also carried out under human- like grasping condition by placing a silicon material with the same properties of prosthetic cosmetic gloves in between the sensor and the inter-element in order to simulate the human skin. Data show that the input–output relationship of the analyzed, sensor is strongly influenced by both the loading condition (i.e., type of inter-element) and the grasping condition (with or without the silicon material). This needs to be taken into account to avoid significant measurement error. To go over this hurdle, the sensors have to be calibrated under each specific condition in order to apply suitable corrections to the sensor output and significantly improve the measurement accuracy.

## 1. Introduction

The human hand is the main interfacing tool between human beings and environment; it enables exploration of the world and learning thanks to perception. Human grasping and manipulation capabilities, as well as discrimination of object physical properties and tactile scanning of a surface are essential for the execution of activities of daily living (ADLs).

The distribution of the forces applied on object surfaces during grasping and manipulation is of paramount importance for studying the quality of a grasp performed by human, robotic or prosthetic hands [1,2,3]. Force and pressure data in grasping tasks are usually collected through two main approaches. The former is grounded on wearable solutions, e.g., sensors integrated in gloves [4,5] or directly positioned on the hand [6]; the latter resorts to the use of instrumented objects [7], e.g., common objects of daily life instrumented with tactile or force sensors.

Wearable solutions suffer from some drawbacks, such as (i) time consuming procedure for sensor positioning and calibration; (ii) a complex wearability, due to a not perfect matching between the sensors and the corresponding anatomical parts; (iii) alteration of natural movements. Therefore, especially when the aim is to analyze performance of hands with different kinematic structures, such as human and prosthetic hands, the use of gloves or else sensors directly applied on the hands could not represent the best solution for grasping analysis.

On the other hand, instrumented objects offer the possibility to measure grasping forces independently on the hand characteristics, e.g., right or left hand, natural, prosthetic or robotic hand.

The forces applied at the contact point on the object surface are characterized by normal and tangential components. Nevertheless, most information about grasping can be extracted by the sole normal component, which is easier to measure. For instance, (a) several force-and-slippage control strategies rely on the sole normal force component [8,9]; (b) object slippage can be retrieved by the normal force [10] and can be adopted for performing a stable grasp by online compensating for possible object slippage [11]; (c) performance indicators in the literature for evaluating the grasp quality use only the normal force [12]; (d) finally, several instrumented objects embed sensors measuring only on the normal component of the applied force, e.g., [13,14,15].

Therefore, this work is focused on an in-depth experimental analysis of capacitive sensors potentially suitable for instrumenting objects for evaluating the normal component of the applied force. Over the years, several technologies have been adopted for developing instrumented objects. The most significant of them are load cells [16], strain gauges [7] piezoresistive sensors, such as Force Sensing Resistor (FSR) sensors [13,14]. Also ad hoc developed sensor modules based on resistive measurements have been used, where the resistance of a conductive elastometer is changed by the applied normal force [15,17]. However, it is shown that the capacitive sensing principle has better performance than other technologies in terms of simple structure, stability and temperature independence [18,19]. This is why this paper is focused on capacitive sensors, in particular Novel GmbH [20] capacitive sensors belonging to the Pliance system (i.e., a Novel product for the analysis of pressure distribution on all kinds of contact surfaces). They are commercially available sensors that address requirements coming from grasping analysis and successfully used in the literature as pressure sensors for biomechanical applications. Thanks to their metrological properties, the flexibility and the wireless data acquisition system, these sensors have already been used in several fields, such as: prosthetics (for measuring pressure distribution on the stump by the socket [21]), rehabilitation (for evaluating dynamic plantar pressure distribution in hemiparetic patients [22]), sport (for monitoring the load on the horse back to define different rider positions [23]) and diagnosis (for studying the effects of obesity in children by monitoring the plantar pressure distributions [24], or in patients with a plantar forefoot ulcer or diabetes [25]). The applications described previously are not studies on the feasibility of these sensors for analyzing the force normal component during grasping and manipulation.

The accuracy, reliability and sensitivity of some models of Novel capacitive sensors have been analyzed in the previously listed applications. In [26], the accuracy of the Pedar in-shoe system (i.e., a Novel product measuring the interaction between foot, shoe and ground, by means of a sensor placed into the shoe) has been investigated. The authors tested the absolute accuracy of the device during the gait cycle, showing that (i) the type of footwear influences the data recorded by the Pedar system; (ii) the estimated load acting on the plantar surface is generally lower than the reference one implying an underestimation of the force magnitude (mean difference 13.4%).

Murphy et al.’s study [27] examined the reliability of the Pedar sensor system showing a reliability level not always acceptable, demonstrated by an intra-class correlation coefficient from 0.529 to 0.762. In addition, in [23] the validity and repeatability of a saddle Novel’s pressure device, in order to evaluate a horse rider position, have been examined. The first one was defined by means of the correlation coefficient between the weight of the rider and the measured normal force, whilst the second one by calculating intra-class correlation coefficients. The obtained results showed that, during the system use, the sensitivity of the sensors changed over time, needing a daily recalibration phase, and the repeatability of the measures decreased with its removal and replacement. This is proved by a daily sensor variation from 4.4% to 5.0%. In [28] a correlation between the force recorded by the device and the texture of the material interposed between the sensor and the load has been observed, showing differences in pressure distribution depending on a rigid or a soft contact area.

The positive results reported in the literature and the lack of studies about the application of Novel sensors for grasping and manipulation force analysis encouraged to investigate their performance and usage in this application. Special attention has been paid to the sensor performance in static conditions; a contact between the load and the sensor has been mediated by an inter-element (called in the following probe). This solution allows overcoming the concerns about reliability and dependency of sensor response on contact characteristics [28]. The probe allowed applying the load over the whole sensor active region, regardless of the specific contact area and the contact material, i.e., the human skin or the prosthetic glove. Given the plurality of situations during grasping that can affect sensor performance, different probes have been developed in order to facilitate the application of the force in the specific sensing regions involved in the targeted grasp. The effect of different shapes of the probe on the input-output relationship has been studied. In addition, human-like grasping condition has been mimicked by using a silicon material (typical of prosthetic cosmetic gloves) reproducing the softness of human skin to evaluate the sensor performance in a scenario close to the application of interest. This aspect is considered of paramount importance in several medical applications [29].

The paper is structured as follows. In Section 2 the requirements to be fulfilled by the sensors have been identified. Then, the main tools and the protocol used to achieve the desired goal are described. Section 3 is focused on the experimental results obtained by processing the pressure data from the sensors and through a comparison with reference values; Section 4 presents critical considerations about the obtained results; finally, in Section 5 conclusions and future work are discussed.

## 2. Materials and Methods

The requirements that a tactile sensor should fulfil for our application (i.e., the analysis of grasping forces in natural and prosthetic hands) are: (i) a measuring range up to 10 N and a discrimination threshold of 0.2 N [30], by referring to the human touch; (ii) a good accuracy of the measure, at least 5% of full-scale range; (iii) a flexibility of the sensor material for its high adaptability to objects of any shape.

A commercial system that satisfies the aforementioned requirements is represented by the Novel GmbH capacitive sensors [20]. They are characterized (from the datasheet [20]) by (i) a measuring range with a discrimination threshold ranged from 0.5 kPa up to 20 kPa, depending on the specific calibration phase fulfilled by the manufacturer (these values correspond to 0.05 N and 2 N respectively); (ii) a steady state ranged between 60 kPa and 2000 kPa (corresponding to 6 N and 200 N respectively); (iii) an accuracy of 5% of full-scale range; (iv) a square active area of the sensing element of 1 cm^2^; (v) high flexibility and different sensor dimensions available that allow us to use them in various configurations and on objects of different shapes.

**Figure 1:**
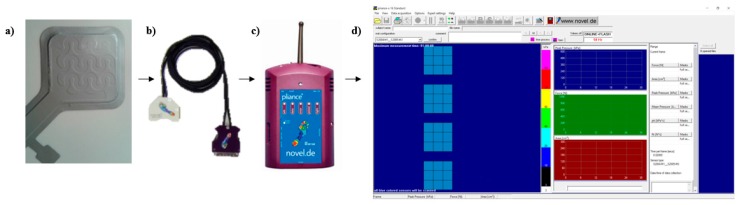
Experimental set-up for data acquisition from the sensor: (**a**) One Socket Sensor XL S2004 and his wave-shaped regions connected through (**b**) a cable to (**c**) a Novel box for the signal acquisition and processing. Data have been collected and sent to a computer. (**d**) Software interface.

The response of four Novel sensors, named in the following 058, 059, 060, 061, model Socket Sensor XL S2004 (Figure 1a) under different loading conditions has been experimentally assessed. These sensors consist of a capacitance transducers matrix whose single sensory element has an area of 1 cm^2^. Each element consists of a hollow wave-shaped active region and a raised wave-shaped inactive one. The sensor presents 9 sensory elements organized in a 3 × 3 matrix. The measuring range of the sensory element is 3 kPa–200 kPa (0.3 N–20 N). Each sensor has been connected to a Novel box (Figure 1c) for the signal acquisition and processing through a connection cable (Figure 1b), which enables data elaboration and communication. Data are transmitted from the Novel box to a computer by means of a Bluetooth communication enabled by a communication key. Data have been collected using a sampling frequency of 50 Hz and have been sent to a computer by means of a Bluetooth communication. The pliance-x online 16 Expert software (Figure 1d shows the software interface) has been used to record and plot contact area, total pressure and total force. The total force $F_{m}$ measured by the sensor matrix at a given time t is computed as

(1) $$F_{m}=A\sum_{i=1}^{n}P_{i},$$

where *A* is the area of each sensing element (i.e., 1 cm^2^), *P_i_* is the pressure value measured by the *i*-th sensing element at time instant t, *n* is the number of sensing elements composing the sensor matrix.

**Figure 2:**
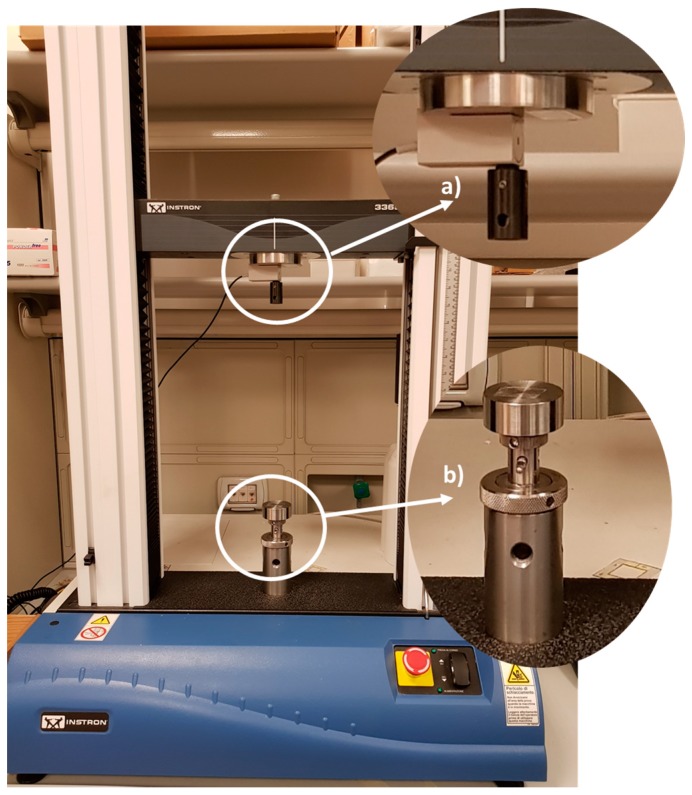
The traction/compression testing machine Instron 3365. With white circles are highlighted: (**a**) The load cell to which the probe is applied; (**b**) The flat support for sensor positioning.

The sensors metrological properties have been estimated by applying forces in the whole range of interest (i.e., up to 9 N) using a traction/compression testing machine, i.e., the Instron 3365 (Figure 2). This system has been equipped with a load cell (measuring forces up to 10 N, accuracy of ±0.25% of the read value) which provided the reference value of the applied force ($F_{r}$).

**Figure 3:**
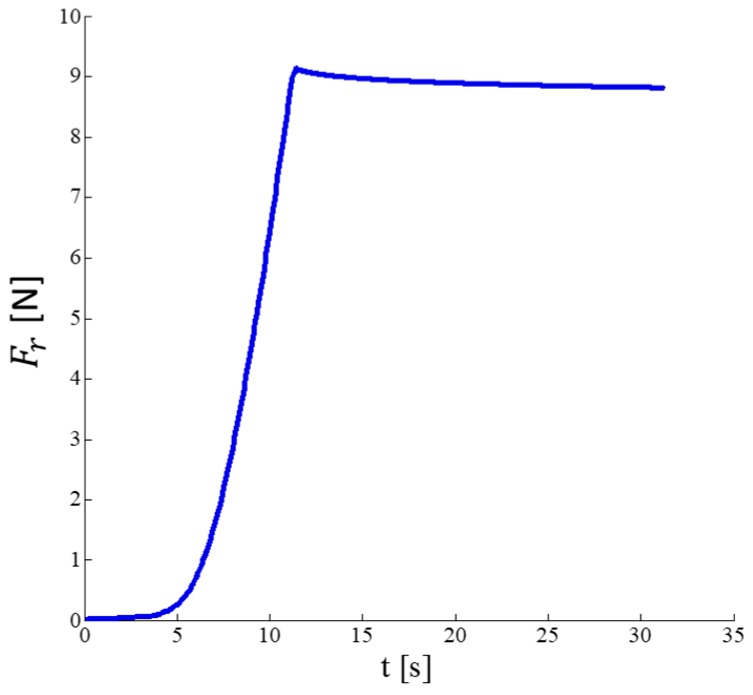
Evolution over time of the force applied on the sensor by using the traction/compression testing machine during the three repetitions. The depth of the curve represents the mean value of the force applied during the three trials ± the standard deviation.

The force has been applied by setting two parameters. The first one is the load speed, set to 2 mm∙min^−1^. The second one is the maximum value of the force to be applied by the Instron during the experiment. Force values from 1 N to 9 N in order to assess the sensor response in the whole range of interest. At the end of each experiment, the load applied on the sensor has been acquired for 10 s during the plateau (with reference to Figure 3, the force has been considered in the interval from 15 s to 25 s) to record the steady-state response of the sensor. Figure 3 shows a typical trend of the applied force $F_{r}$ with a maximum value of 9 N. For each value, three acquisitions were made under repeatability conditions [31]. The low number of trials has been taken into account for computing the coverage factor to estimate the expended uncertainty: the lower the number of trials the bigger the coverage factor [31]. Indeed, a t-student distribution with 2 degrees of freedom (number of trials −1) has been used. In Figure 3, the depth of the curve represents the mean value ± the standard deviation of the force recorded during the three trials. Due to the high repeatability in the measure, the standard deviation value is low (i.e., the maximum calculated value is about 0.07 N).

**Figure 4:**
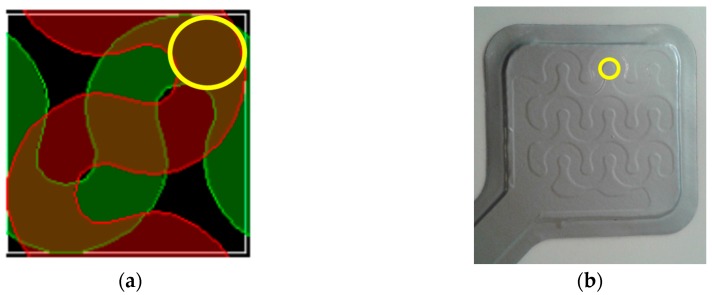
(**a**) Internal structure of a sensing element of the sensor XL S2004. The most active region, where two sensing elements are superimposed, is encircled in yellow; (**b**) External view of the sensor with the most active region encircled in yellow.

The sensor has been tested in three different loading conditions: (A) Distributed load; (B) 9 Tips load; and (C) Wave-shaped load. As shown in Figure 1a, the sensor is composed of 9 sensory elements organized in a 3 × 3 matrix with hollow wave-shaped active regions and raised wave-shaped inactive ones. First of all, the load has been applied on the whole sensor surface (i.e., “Distributed load” loading condition), in order to test the sensor in a condition comparable with a finger applying a force on the sensor. Inspecting the internal structure of each sensing element (Figure 4a), an area can be observed, where two sensing elements are superimposed (it is encircled in yellow in Figure 4a). This area represents the most active region of the sensor. Therefore, the load has been applied on the 9 most active regions of the 9 sensory elements of the sensor by means of “Tips load”. Finally, in order to apply the load on the whole wave-shaped active region, a “Wave-shaped load” loading condition has been also tested.

**Figure 5:**
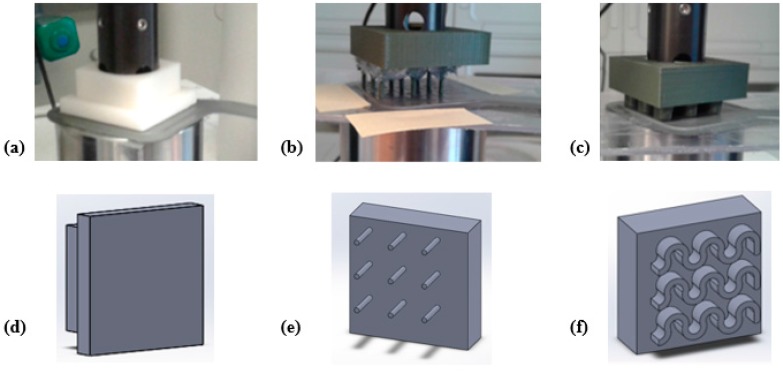
Experimental procedure of coupling between the sensor and the loading probe employed for the three configurations: (**a**) Distributed load; (**b**) 9 Tips load; (**c**) Wave-shaped load. In (**d**–**f**) is shown the 3D CAD for the three configurations.

To this purpose, ABS (Acrylonitrile butadiene styrene) probes have been purposely developed with a 3D printing technique. In the Distributed load, the load has been applied on the sensor in a distributed way by means of a probe with a flat surface shown in (Figure 5a,d). The loading area had dimensions of 30 × 30 mm^2^ corresponding to the sensor active area. In 9 Tips load, the load has been applied using a probe consisting of 9 tips with height of 10 mm, diameter of 1.1 mm and edge-to-edge distance of 10.1 mm (Figure 5b,e). This element allowed applying the load in correspondence of the most sensitive areas on each sensing element of the matrix. In Wave-shaped load, the probe used in the third configuration presented a wave-shaped loading region that matches with the active region of the sensor (Figure 5c,f). Therefore, these three probe shapes allowed testing the most significant loading conditions.

**Figure 6:**
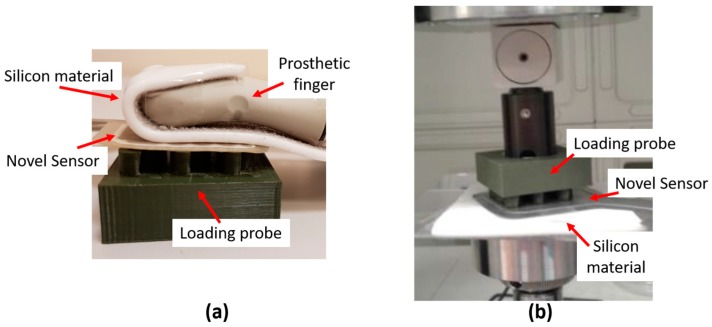
Human-like grasping condition. The real condition (**a**) is mimicked with the setup shown on the right (**b**).

In order to replicate human-like grasping condition, the contact between the human fingertip and the sensor has been mimicked by positioning a silicon material on the sensor surface opposite to the probe (Figure 6). The material, taken from a cosmetic glove used in commercial prosthesis (i.e., the Steeper silicone cosmetic glove [32]), wants to emulate the softness of human skin. In Figure 6a, the real grasping condition is shown: the prosthetic finger, covered by the silicone cosmetic glove, is in contact with the sensor positioned on the loading probe placed on the object to be grasped. The mimicked version of the real grasping condition is shown in Figure 6b, where the prosthetic finger is simulated by the rigid component under the silicone material. The experiments were performed under the two loading conditions that reached the best performance (i.e., 9 Tips load and Wave-shaped load), as shown in Section 3.

In order to guarantee the reproducibility of the results, for a single configuration, the experiments have been performed under repeatability conditions (same measurement procedure, same operators same measuring system and same location, as recommended in [33]). In addition, measurements have been performed changing conditions of use (loading conditions in terms of probe shape and the presence or not of the silicon material).

The input–output relationship of the sensor under test has been obtained by means of a linear fitting between $F_{r}$ and $F_{m}$ in the whole range of interest, considering the three aforementioned loading conditions, and the grasping conditions (with or without the silicon material). The best fitting curve for each condition has been calculated by applying the mean square error algorithm.

A further analysis has been performed to quantify the difference between $F_{r}$ and $F_{m}$ by using the Bland Altman analysis [34]. This analysis is largely used to compare the output provided by a measuring system under test with the one provided by a reference system. In particular we represented the Bland Altman plot, that shows on the x-axis the mean value of the measurements obtained by the two systems (in our case $\bar{F}$) and on the y-axis the difference between the values provided by the two systems (in our case ΔF). Both the mean of difference (MOD, that is the mean of all the values reported on the x-axis) and the limits of agreement (LOA, calculated as MOD ± 1.96∙SD, where SD is the standard deviation of all the values reported on the y-axis) have been calculated. This analysis has been performed considering two conditions: (i) the direct comparison between $F_{r}$ and $F_{m}$; (ii) the comparison between $F_{r}$ and the force $F_{c}$, which represents a correction of $F_{m}$. $F_{c}$ is computed with two analytical steps: firstly, the linear fitting curve between $F_{r}$ and $F_{m}$ is inverted and two interpolation coefficients are extracted; secondly, the coefficients are inserted into a linear relationship between $F_{m}$ and $F_{c}$.

Differences between experimental data obtained using a distributed load and results obtained under all the other conditions (9 Tips load, and Wave-shaped load, with and without the silicon material) were compared using Student’s paired *t*-test and were considered significant for *p*-value < 0.05.

Matlab^®^ software (MathWorks, Natick, MA, USA) package has been used to analyse the experimental data collected during the experiments.

## 3. Results

Results show similar behavior among the four Socket Sensor XL S2004 (i.e., 058, 059, 060, 061) with a maximum difference among them of 0.4 N–0.5 N in correspondence of an applied load of 9 N. Therefore, for the sake of brevity, only the data about one of these sensors (i.e., S2004–060) are reported in the following.

**Figure 7:**
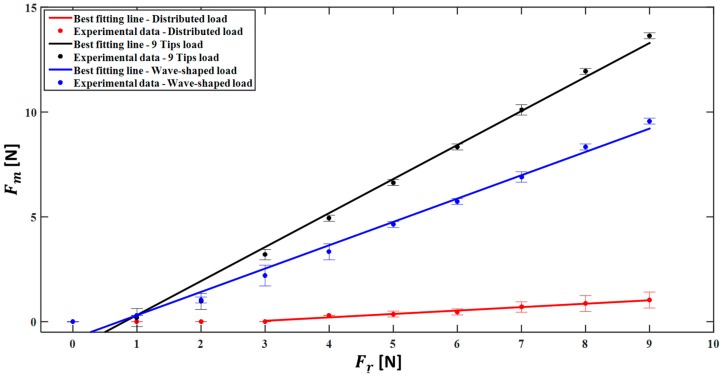
Mean value of force measured ± expanded uncertainty, for the sensor S2004–060 and each reference load applied. The best fitting line for each examined loading condition is also shown.

Figure 7 shows the relationship between $F_{r}$ and $F_{m}$ for the Socket Sensor XL S2004–060. $F_{m}$ values are reported as mean value ± expanded uncertainty over the three repeated trials for each applied load (e.g., for an applied load of 9 N, the following values of $F_{m}$ were measured: 1.03 ± 0.15 N for the Distributed load, 13.63 ± 0.06 N for the 9 Tips load, 9.57 ± 0.06 N for the Wave-shaped load). The expanded uncertainty has been estimated considering a Student reference distribution with 2 degrees of freedom and a level of confidence of 95%, as recommended in [31]. In Figure 7, the error bars represent the confidence intervals considering the expanded uncertainty calculated using the t-student reference distribution.

The best fitting line representing the sensor calibration curve has been calculated considering $F_{r}$ bigger than the discrimination threshold (i.e., 4 N in the case of the Distributed load, 1 N for both the 9 Tips load and the Wave-shaped load).

The results show that, in general, the sensor response was strongly influenced by the loading condition A, B or C. In fact, the discrimination threshold ranges from 1 N (for the Wave-shaped load and the 9 Tips load) to 4 N (for the Distributed load). Considering only the force values discriminated by the sensor (from 4 N to 9 N in the case of the Distributed load, from 1 N to 9 N for the 9 Tips load, from 1 N to 9 N for the Wave-shaped load), the relationship between $F_{r}$ and $F_{m}$ is well represented by a linear model, as confirmed by the high value of the correlation coefficient (*r*^2^) reported in Table 1 (i.e., 0.980, 0.986, 0.988, for the Distributed load, 9 Tips load and Wave-shaped load, respectively). The sensitivity, considered as the slope of the best fitting line, is 0.1631, 1.622, 1.113 for the three loading conditions, respectively.

**Figure 8:**
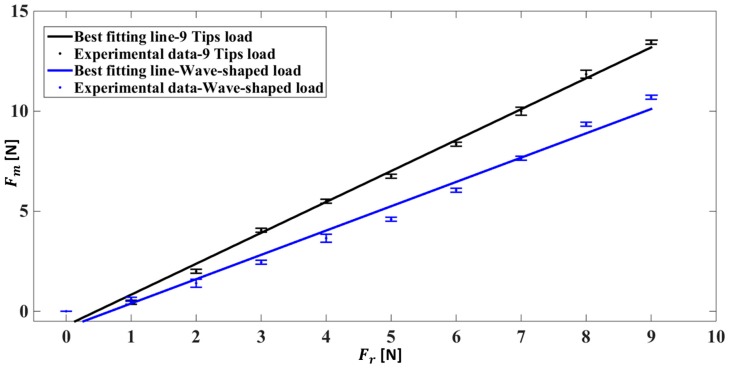
Mean value of force measured ± expanded uncertainty, for the sensor S2004–060 under human-like grasping conditions and each reference load applied. The best fitting line for each examined loading condition is also shown.

The calibration has been performed under human-like grasping conditions in the two cases of 9 Tips load and the Wave-shaped load (i.e., loading conditions with the highest sensitivity), as shown in Figure 8.

Also in this case the loading conditions influence the input–output relationship of the system in terms of discrimination threshold and sensitivity. In order to have a clear picture of the assessed metrological properties in all the different analysed conditions, the sensitivity and the discrimination threshold are summarized in Table 1.

**Figure 9:**
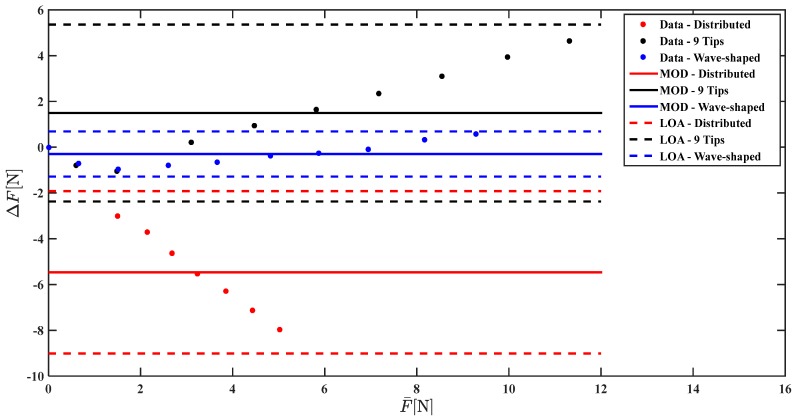
Bland Altman analysis comparing $F_{m}$ with $F_{r}$ for the three loading conditions (data related to Distributed load, 9 Tips load, and Wave-shaped load are shown in red, in black, and in blue, respectively).

**Figure 10:**
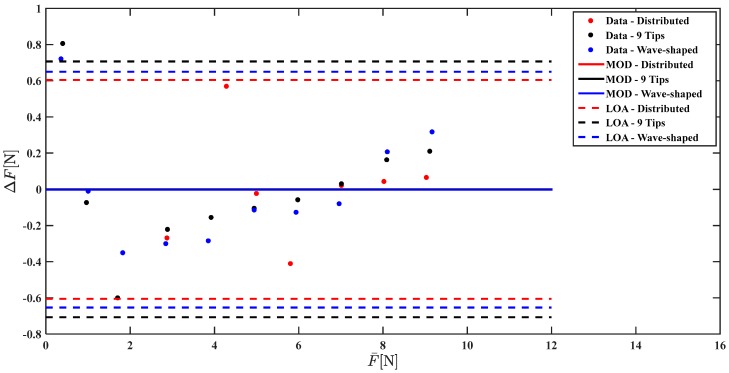
Bland Altman analysis comparing $F_{c}$ with $F_{r}$ for the three loading conditions (results obtained with the Distributed load, with the 9 Tips load and with the Wave-shaped load are shown in red, in black and in blue, respectively).

Figure 9 and Figure 10 show the Bland Altman analysis that allows the comparison of $F_{m}$ and $F_{c}$ with $F_{r}$.

The Bland Altman analysis shown in Figure 10 points out a marked underestimation of the force applied when the Distributed load is used (MOD = −5.47 N), a slight underestimation using Wave-shaped load (MOD = −0.2 N), and a marked overestimation using the 9 tips load (MOD = +1.8 N). The agreement between the reference force values and the measured ones largely improve when a correction based on a linear model is used; indeed the MOD is almost null considering all the three loading conditions (Figure 10).

**Figure 11:**
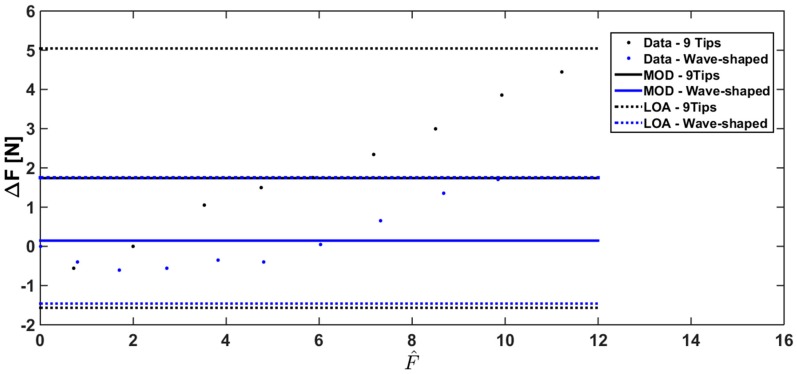
Bland Altman analysis comparing $F_{m}$ with $F_{r}$ for the two loading conditions under human-like grasping conditions (data related to 9 Tips load, and Wave-shaped load are shown in black and in blue, respectively).

**Figure 12:**
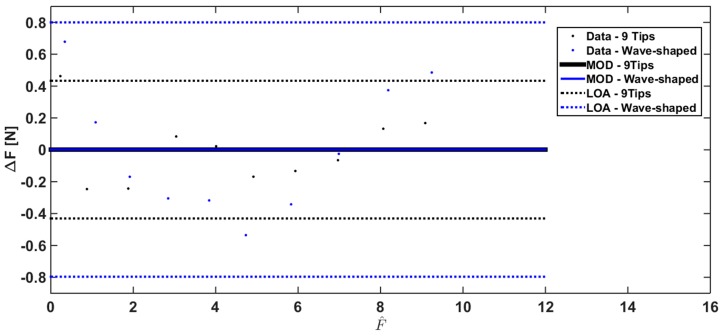
Bland Altman analysis comparing $F_{c}$ with $F_{r}$ for the two loading conditions under human-like grasping conditions (results obtained with the 9 Tips load and with the Wave-shaped load are shown in black and in blue, respectively).

The Bland Altman analysis has also been carried out under human-like grasping conditions (Figure 11 and Figure 12) to investigate sensor performance in a more realistic scenario (i.e., with the silicon material on the sensor surface opposite to the probe, emulating the human skin).

The Bland Altman analysis shown in Figure 12 points out a slight overestimation using Wave-shaped load (MOD = +0.145 N), and a marked overestimation using the 9 tips load (MOD = +1.75 N). The overlap between the reference force values and the measured ones largely improves when a correction based on a linear model is used; indeed the MOD is almost null considering the two loading conditions (Figure 12).

Forces measured under the four loading conditions (9-Tips with and without silicon material, wave-shaped with and without silicon material) were significantly higher than the ones measured when a distributed load is applied (*p*-value < 0.05).

## 4. Discussion

The performed analysis aimed at (i) quantifying the influence of specific loading conditions (which can be used during the evaluation of grasping tasks in a real scenario) on sensor response; and (ii) understanding how the use of specific loading conditions may improve the sensor performance for the considered application (i.e., grasping and manipulation force analysis). Although these commercial sensors have been assessed in several biomedical fields (such as measuring pressure distribution between the stump and the socket in prosthetics [21], rehabilitation [22], sport [23] and diagnosis [24,25]), to the best of our knowledge, this is the first study focused on the response of these sensors for grasping and manipulation force analysis. The effect of a probe on sensor performance has been briefly analyzed in a authors’s previous work [14], but using a different type of sensors (i.e., piezoresistive). This study is focused on capacitive sensors because of their better performance with respect to other technologies [18,19].

The obtained results showed that, in order to use the analysed sensors for the specific application of our interest (grasp forces analysis), it is necessary to take into account the loading conditions (i.e., the shape of the probes and the type of material the sensor is in contact with), since it strongly influences the sensor response. In fact, the results shown in Figure 7 demonstrated that the sensors cannot be used with satisfactory performance when a distributed load (condition comparable with a finger applying a force on the sensor) is applied on the sensor (better discrimination threshold and sensitivity are required).

Experiments without any material interposed between the probe and the sensor were performed under three different conditions. When a Distributed load is applied, the sensor response is linear, but it underestimates the force as testified by a sensitivity < 1 (i.e., 0.163) and a MOD < 0 (i.e., −5.47 N), and the discrimination threshold is >3 N. Indeed, under this configuration the force is uniformly applied on the whole sensor and not only on its sensing part. Therefore, the lack of sensitivity and the high discrimination threshold implies that this configuration does not fulfill the requirements listed in Section 2. Using a correction based on a linear model, the estimated force is very close to the reference value in all the measurement range, without a substantial underestimation (as shown by the low value of the MOD, see Figure 8); however, the discrimination threshold cannot be improved. The results obtained with the 9 Tips load showed a remarkable increase of sensor sensitivity (i.e., 1.62 and 1.55 in the human-like grasping condition). In fact, when a load close to 9 N is applied, the recorded force is significantly higher than the actual one. In addition, this solution allows improving the discrimination threshold (i.e., 1 N) which is close to our requirements. This is caused by the punctual load applied on each of the nine sensing elements of the sensor in correspondence of the most sensitive region. Although this loading condition introduces an overestimation of the applied force, it can be corrected by using the linear model calculated through the calibration of the sensor. The Wave-shaped probe replicates the sensor structure and allows obtaining a good discrimination threshold (i.e., 1 N), comparable to the 9 Tips load, and high sensitivity (i.e., 1.11 and 1.22 in the human-like grasping condition). Also in this case the correction allows improving the accuracy. Therefore, specific loading conditions may improve the aforementioned metrological properties, and the calibration curve obtained for the specific condition has be used to avoid significant measurement error.

In addition, the sensors were assessed under human-like grasping conditions (by interposing a silicon material between the probe and the sensor). From the obtained results, we can conclude that the metrological properties of the Socket Sensor XL S2004, in particular discrimination threshold and sensitivity, also depend on the presence of the silicon material.

In the context of grasping analysis performed by instrumented objects, the loading area depends on a multitude of factors: the type of the object, the hand characteristics and the specific task to perform. Due to the significant influence of the loading area of the Socket Sensor XL S2004, this system may be affected by relevant measurement error. The use of a probe placed between the object and the load guarantees a specific loading area regardless the previously listed factors. Therefore, the proposed solution may overcome these concerns during grasp analysis by associating a specific calibration curve to the specific condition, such as probe shape and interposing material. Furthermore, depending on the specific application, it is possible to converge toward the loading solutions of 9 Tips load or Wave-shaped load (if a high sensitivity and low discrimination threshold is required). These findings aim at maximizing the performance of the Socket Sensor XL S2004 for the considered application (i.e., grasping and manipulation force analysis).

## 5. Conclusions

In this paper, the feasibility of using a commercial capacitive pressure sensor for instrumenting objects and performing grasp analysis has been investigated. Firstly, performance of the sensor has been analyzed in function of different loading conditions. Then, the sensor assessment has been performed under human- like grasping conditions, by emulating skin contact though silicon material.

The use of calibration curves specific for each configuration, taking into account both the loading conditions and the material interposed between the sensor and the probe, allows improving the accuracy of the sensor, as well as modulating its sensitivity and discrimination threshold.

The probes proposed in this paper lead to create a system for a reproducible measure also for the targeted application, aiming to measure (through an instrumented object) the contact force applied by human or prosthetic hands during grasping and manipulation tasks independently of the contact material, the loading area, the hand characteristics and the specific task.

Future work will be devoted to use the obtained results for developing instrumented objects for the analysis of grasping and manipulation. These devices can enable quantitative and objective evaluations of grasp and manipulation capabilities of the hand, and are adaptable to any subject regardless of the specific anthropometric characteristics. It is worth noting that other influencing factors may be investigated, such as the influence of sensor bending during different grasping tasks. Finally, as further step, the development of instrumented objects with these sensors will be carried out in order to study grasping capabilities in ADL tasks in healthy subjects and amputees wearing prosthetic hands.

## Figures and Tables

**Table 1:** Results obtained from the calibration curve for each loading condition, considering the sensor S2004–060.

Loading Condition	Sensitivity	Discrimination Threshold	*r*^2^
Distributed load	0.163	4 N	0.980
9 Tips load	1.62	1 N	0.986
Wave-shaped load	1.11	1 N	0.988
9 Tips load-Silicon	1.55	1 N	0.995
Wave-shaped load-Silicon	1.22	1 N	0.982
